# The Clinical Diagnosis-Based Nationwide Epidemiology of Metabolic Dysfunction-Associated Liver Disease in Korea

**DOI:** 10.3390/jcm12247634

**Published:** 2023-12-12

**Authors:** Nam-Hun Lee, Seok-Ju Jeong, Jing-Hua Wang, Yu-Jin Choi, Hyeon-Muk Oh, Jung-Hyo Cho, Yo-Chan Ahn, Chang-Gue Son

**Affiliations:** 1 East-West Cancer Center, Cheonan Korean Medical Hospital, Daejeon University, 4 Notaesan-ro, Seobuk-gu, Cheonan-si 31099, Republic of Korea; nhlee@dju.ac.kr; 2 Health Insurance Review & Assessment Service, Dunsanbuk-ro 121, Seo-gu, Daejeon 35236, Republic of Korea; kju@hira.or.kr; 3 Liver-Immunology Research Center, Daejeon University, 176 Daedeok-daero, Seo-gu, Daejeon 35235, Republic of Korea; ewccwang@gmail.com (J.-H.W.); chyj433@naver.com (Y.-J.C.); oh033@naver.com (H.-M.O.); choajoal@dju.kr (J.-H.C.); 4 Department of Health Service Management, Daejeon University, 62 Daehak-ro, Dong-gu, Daejeon 35620, Republic of Korea; ycahn@dju.kr

**Keywords:** MASLD, potential underestimation of prevalence, nationwide dataset, HIRA records, sex-specific differences

## Abstract

Background: Although most epidemiological studies have been conducted using a relatively small population or subjects who had medical screenings, the present study aimed to investigate the incidence and prevalence of MASLD (formerly NAFLD) in Korea using nationwide registry data provided by the Health Insurance Review and Assessment Service (HIRA). Methods: Using nationwide medical records provided by HIRA, we analyzed the entire dataset of patients with MASL (KCD10-K76.0) and MASH (KCD10-K75.8) from 2010 to 2021 and calculated the incidence and prevalence by year, age, and gender. The prevalence and incidence rates were calculated by analyzing the HIRA data covering almost the entire population of Korea for 12 years, from 2010 to 2021, with an average population of 50,856,244 during this period. Statistical analyses included calculating confidence intervals using Ulm’s formula and conducting sex- and age-specific analyses with a Cochran–Armitage test for trends. Results: The annual incidence of MASL/MASH increased significantly from 9.71/0.37 in 2010 to 13.95/5.52 per 1000 persons in 2021 (*p* < 0.01). The annual prevalence of MASL increased from 15.69 in 2010 to 34.23 per 1000 persons in 2021, while the annual prevalence of MASH increased from 0.49 to 9.79 per 1000 persons between 2010 and 2021 (*p* < 0.01). Regarding the sex-dimorphic feature of MASLD, there was a male predominance in those < 50 years old but a female predominance in those ≥ 50 years old for the incidence and prevalence of MASL and the incidence of MASH. Conclusion: The incidence of MASL increased by 3% to 4% every year, while the incidence of MASH increased 14.91-fold from 2010 to 2021. The increasing trend is noteworthy compared with previous reports.

## 1. Introduction

A fatty liver is generally classified as an alcoholic- or metabolic dysfunction-associated steatotic liver (MASL, formerly NAFL), depending on the extent of alcohol in the pathogenesis of hepatosteatosis [1]. Along with the consistent increase in the obese population and changes in lifestyles, metabolic dysfunction-associated liver disease (MASLD, formerly NAFLD) is attracting increasing attention as a major public health problem worldwide [2]. MASLD is now known as a key contributor to not only hepatic diseases but also various extrahepatic disorders, such as type 2 diabetes or cardiovascular diseases [3,4].

MASLD refers to a wide spectrum of diseases from mere steatosis without inflammation (MASL) to metabolic dysfunction-associated steatohepatitis (MASH, formerly NASH), liver fibrosis, cirrhosis or hepatoma [5]. MASLD is strongly associated with insulin resistance, which is involved in its pathogenesis and progression to MASH [6]. According to a study conducted on Koreans, individuals with non-alcoholic fatty liver disease had a significantly higher body mass index (22.3 kg/m^2^ versus 25.4 kg/m^2^) and a significantly higher prevalence of diabetes (3.7% versus 9.4%) and metabolic syndrome (10.7% versus 41.4%) compared to the control group [7]. Regarding the pathophysiologic aspects of MASLD, the progression from MASL to MASH is an important step that increases the risk of the development of fibrosis, cirrhosis and hepatocellular carcinoma (HCC) [8]. A previous meta-analysis reported a 15-fold higher liver-specific mortality among patients with MASH than among those with MASL [9].

On the other hand, the epidemiology of MASLD is affected by ethnic background, sex, age, and environmental factors [10]. The global prevalence of MASLD is approximately 32.4% [9], and that of MASH ranges from 3% to 5% [9]. Age is generally a risk factor for the development of MASLD and MASLD-related fibrosis [11]. The male predominance of MASLD is thought to be exchanged with female predominance after menopausal age, but a study in Thailand reported a female prevalence regardless of menopausal age [12,13]. South Korea is one of the countries that has experienced rapid urbanization. Our previous systematic analysis found that the prevalence of MASLD was 30.3% and was continuously increasing in Korea [14]. However, most epidemiological studies have been conducted using a relatively small population or subjects who had medical screenings.

The present study aimed to investigate the real-world data for the incidence and prevalence of MASLD in Korea based on clinical diagnoses by physicians. We herein conducted an analysis using nationwide medical records provided by the Korean government through the Korean Health Insurance Review and Assessment Service (HIRA).

## 2. Materials and Methods

### 2.1. Patients and Study Design

The present retrospective population-based study aimed to investigate the incidence and prevalence of MASLD, including MASL and MASH, among Koreans through a complete enumeration survey using HIRA claims data from 1 January 2010, to 31 December 2021. Korea has a national health insurance system that provides medical insurance coverage to all its inhabitants, and each individual is registered with a health insurance identification number. Every resident is eligible regardless of nationality or profession. The system is funded by compulsory contributions from all residents and government subsidies. All Korean patients who visit a hospital are assigned a diagnosis according to the Korean Classifications of Diseases, 10th Revision (KCD10), a modification or adaptation of the International Classifications of Diseases, 10th Revision (ICD10). The HIRA reviews the data to ensure proper diagnoses and management and to assess the quality of health care and medical fees.

To determine the incidence rates of MASL and MASH, we included patients who were newly registered with a diagnostic code of K76.0 according to the KCD10/ICD10 or K75.8 according to the KCD10/ICD10 in either the main or sub diagnosis. To determine the prevalence rates of MASL and MASH, we included patients who were registered with a diagnostic code of K76.0 or K75.8 in either the main or sub diagnosis and documented at least one claim in the corresponding year. It is important to note that for the analysis, liver diseases caused by factors other than MASL or MASH, such as alcohol-related liver disease or other factors, were excluded.

**Figure 1:**
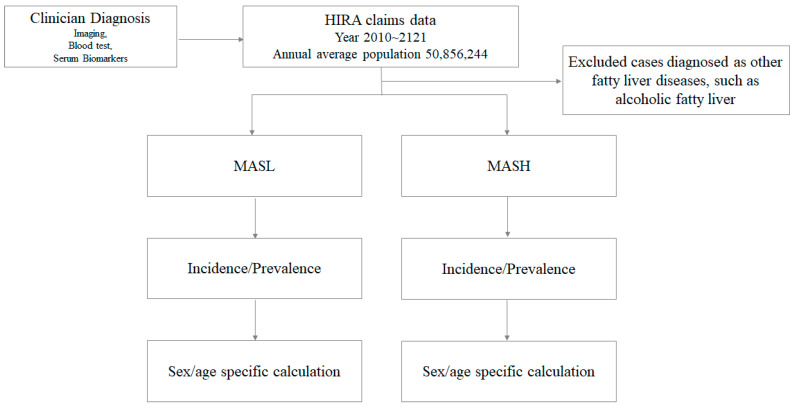
A flowchart outlining the Selection Process for Eligible Study population. Clinician-diagnosed MASL/MASH cases from HIRA claims data were chosen for calculating the incidence/prevalence of MASL/MASH, including sex- and age-specific calculations.

This investigation drew its strength from the comprehensive analysis of HIRA data, covering nearly the entire Korean population over a span of 12 years, from 2010 to 2021. The average population during this period was approximately 50,856,244. To enhance the granularity of our findings, we conducted additional analyses by stratifying the adult population according to gender and age groups (e.g., 20–29 years, 30–39 years, …, 80 years and older) for each respective year. A flowchart outlining the selection process for the eligible study population for the final analysis has been created (Figure 1).

### 2.2. The Diagnosis of MASL and MASH in Clinical Practice

The diagnosis of MASL and MASH in current clinical practice involves a multifaceted approach. While there is not a single universally applied classification model, key diagnostic methods commonly utilized in the clinical setting include noninvasive methods such as imaging techniques (CT, MRI), transient elastography, and blood tests encompassing liver function tests to evaluate liver enzymes, as well as serum biomarkers like the NAFLD Fibrosis Score (NFS) and Fibrosis-4 (FIB-4) index [15]. Additionally, liver biopsy is another diagnostic option. As the present data were extracted from Korean public health insurance records generated by numerous physicians, it was impossible to identify the specific approaches used for diagnosis or classification.

### 2.3. Disclosure of Ethical Statements

This study followed the principles outlined in the Declaration of Helsinki. Approval for this study was obtained from the Institutional Review Board for Human Research of Daejeon University Daejeon Hospital (Protocol number DJDSKH-22-E-10-1, approved on 18 May 2022), which exempted the study from the requirement of obtaining informed consent.

### 2.4. The Calculation of Incidence and Prevalence

Incidence Calculation: The incidence of MASL/MASH was calculated by determining the number of patients who were newly diagnosed with MASL/MASH during a specific calendar year and then dividing this number by the total number of people living in Korea during that same calendar year. This calculation helped to estimate the rate at which new cases of MASL/MASH are occurring in the population. To determine the exact confidence intervals for the incidence, Ulm’s formula was employed. Ulm’s formula is a statistical method used to calculate confidence intervals for incidence rates, which take into account the uncertainty associated with the calculated incidence rate [16].

Prevalence Calculation: The prevalence of MASL/MASH was calculated by dividing the number of patients who were registered with a diagnosis of MASL/MASH during a specific calendar year by the total number of people living in Korea during that same calendar year. This calculation provided an estimate of the proportion of the population that is affected by MASL/MASH. To calculate the 95% confidence intervals for the prevalence, a formula was used: $p\pm 1.96\sqrt{\frac{p(10p)}{n}}$. In this formula, ‘*p*’ represents the prevalence and ‘*n*’ represents the sample size. This method helped to determine the range within which the true prevalence was likely to fall with a 95% level of confidence.

Sex- and Age-Specific Incidence and Prevalence: For a more detailed analysis, the incidence and prevalence of MASL/MASH were calculated separately for different age and gender groups. Population data from the Korean Statistical Information Service in 2015 were used for this purpose. Both the incidence and prevalence were expressed per 1000 inhabitants, allowing for a standardized comparison across different population subgroups. To investigate and compare time trends in incidence by sex and age group, a Cochran–Armitage test for trends was applied. Statistical significance was determined by evaluating the *p*-values, with values less than 0.05 considered statistically significant, indicating trends in incidence over time. This comprehensive approach to data analysis ensured that the study provided a detailed understanding of the incidence and prevalence of MASL/MASH within the Korean population while considering different demographic factors.

### 2.5. Statistical Analysis

A Cochran–Armitage Trend Test was performed using R v4.3.0 and RStudio 2022.07.2 Package. The calculation of the 95% confidence interval (CI) was conducted using the MS-Excel program (2019).

## 3. Results

### 3.1. The Annual Incidence of MASL and MASH in Korea

**Figure 2:**
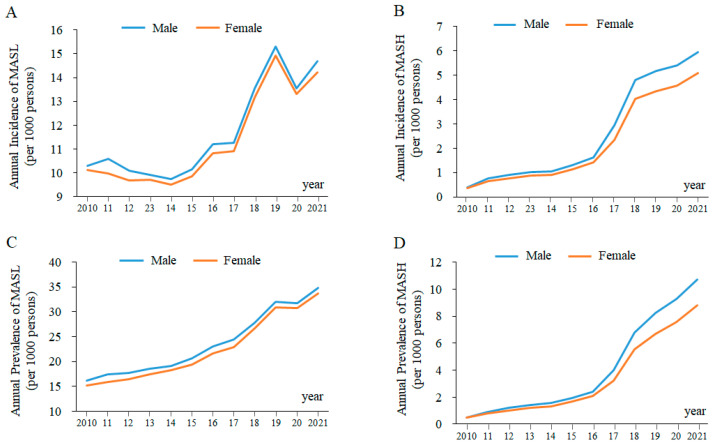
The Annual Incidence and Prevalence of MASL and MASH. The annual incidence of MASL is shown in (**A**) and MASH in (**B**), while the annual prevalence of MASL is presented in (**C**) and MASH in (**D**).

There is an increasing trend in the annual incidence of MASL and MASH in Korea (Figure 2A,B). The overall incidence of MASL during the study period was 11.02 per 1000 persons (male, 11.20; female, 10.85). The annual incidence of MASL increased significantly from 9.71 per 1000 persons in 2010 to 13.95 per 1000 persons in 2021 (*p* < 0.01), and this increasing trend was similar in both males (9.80 in 2010 to 14.18 per 1000 persons in 2021) and females (9.61 in 2010 to 13.72 per 1000 persons in 2021). The incidence increased by approximately 3% to 4% every year from 2010 to 2021 (Table 1).

The annual incidences of MASH during 2010 and 2021 are displayed in Table 2. The overall incidence of MASH over 12 years (2010~2021) was 2.41 per 1000 persons (male, 2.61; female, 2.21). The annual incidence of MASH increased by approximately 14.91-fold from 2010 to 2021, from 0.37 per 1000 persons in 2010 to 5.52 per 1000 persons in 2021 (*p* < 0.01). This rapid increase in MASH incidence showed the same pattern in both males (0.39 in 2010 to 5.94 per 1000 persons in 2021) and females (0.36 in 2010 to 5.10 per 1000 persons in 2021).

### 3.2. The Annual Prevalence of MASL and MASH in Korea

The distribution of the annual prevalence of MASL and MASH is shown in Figure 2C,D. The overall prevalence of MASL in Korea increased significantly from 15.69 to 34.23 per 1000 persons between 2010 and 2021 (male, 16.17 to 34.82; female, 15.22 to 33.64 per 1000 persons). On the other hand, the overall prevalence of MASH increased 19.97-fold from 0.49 to 9.79 per 1000 persons between 2010 and 2021 (male, 0.51 to 10.74; female, 0.47 to 8.84 per 1000 persons). The detailed number of cases of MASL/MASH and prevalence rate according to sex and year are described in Table 3 and Table 4.

### 3.3. Age- and Sex-Related Features of MASL and MASH

**Figure 3:**
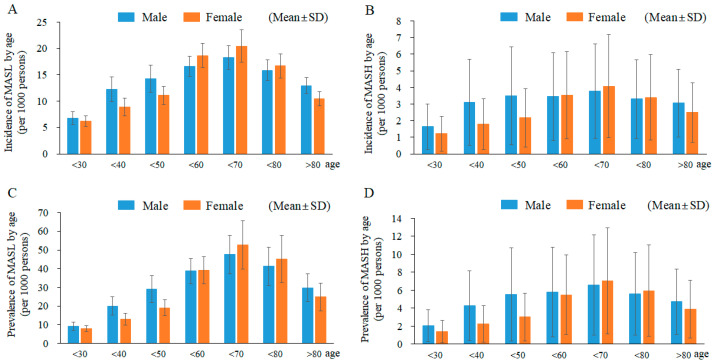
The Incidence and Prevalence of MASL and MASH by age. Newly diagnosed cases of MASL are presented in (**A**) and MASH in (**B**) according to age. The number of individuals treated for MASL is depicted in (**C**) and for MASH in (**D**) for each respective year, organized by age.

The frequencies of MASL and MASH dynamically changed according to aging. The mean rates of both the incidence and prevalence of MASL (incidence, 11.02; prevalence, 23.02 per 1000 persons) and MASH (incidence, 2.41; prevalence, 3.73 per 1000 persons) peaked at 60 to 69 years old in both males and females, which were approximately 2-fold more than the rates at 30 to 39 years (Figure 3A–D, Table 1, Table 2, Table 3 and Table 4). Age- and sex-related annual incidences of MASL and MASH are described in Supplementary Tables S1 and S2. Regarding the sex-dimorphic feature of MASLD, our results showed a male predominance for those < 50 years old but a female predominance for those ≥ 50 years old for the incidence and prevalence of MASL and the incidence of MASH (Figure 3A–D).

## 4. Discussion

South Korea has a national health insurance system that covers the entire population of Korea (51,333,253 persons, 2021), which allows for the collection of accurate medical information related to MASLD [17]. In the present study, we analyzed the nationwide characteristics of patients treated for MASL or MASH (formerly NAFL and NASH) from 2010 to 2021 using Korean government-supported HIRA data that had collected the whole medical diagnosis conducted by Korean medical doctors.

Based on the medical record by HIRA, both the incidence and prevalence of MASL increased by 1.44-fold and 2.18-fold from 2010 to 2021, respectively (Figure 2A,C and Table 1 and Table 3). This increasing pattern is in accordance with other community-based studies; a cross-sectional study showed a 1.5-fold increase in MASLD prevalence between 2015 and 2021 among the Korean military population [18]. Regarding MASH patients, there was a dramatic increase in the incidence and prevalence of MASH over 12 years, 14.92- and 19.98-fold, respectively (Figure 2B,D and Table 2 and Table 4). A US population-based study also shared similar findings with our present data, in which the number of patients diagnosed with MASH increased 12.3-fold between 2010 and 2020 [19]. One systematic review reported 30% of the global MASLD prevalence in 2019 (25% in 2016) and an estimation of MASH between 2% and 6% in the general population [20]. We also previously reported similar prevalence rates, i.e., 30.3% and 2.2%, of MASLD and MASH in Korea using 61 study-derived meta-analyses [14]. These 61 studies, however, obtained the prevalence rates of MASLD through medical screenings conducted on the entire participant population. The prevalence rates reported in the current study were only one tenth of the previous rate reported for MASLD (3.4%) and less than half of the previous rate reported for MASH (1.0%) [21]. The current data do not accurately reflect the prevalence rate of MASLD, but our results show the reality in clinics producing very low diagnosis and treatments, particularly for MASL. These facts may indicate the underestimated prevalence of MASL in the HIRA data due to the fact that MASL is an asymptomatic condition ignored by patients and/or physicians sometimes.

MASH is a more advanced stage of MASLD. The prevalence of MASH increased from 0.49 to 9.79 per 1000 Korean individuals between 2010 and 2021 in the present data (Figure 2D, Table 4). This steep increase in the prevalence of MASH is likely related to the accumulating risk factors, such as changing dietary habits, sedentary lifestyles, and an aging population [22]. Comparing this to MASL, physicians also have been aware of its risk of progressing into hepatic cirrhosis along with better diagnostic capabilities [23]. The prevalence of MASH in the United States also increased from 1.51% in 2010 to 2.79% in 2020 [19]. Approximately 20% of individuals with MASL develop MASH, and over 40% of MASH cases progress to fibrosis [9,24]. Unlike MASL, the presence of MASH directly impacts morbidity and mortality [25]. Accordingly, distinguishing MASL and MASH is very important because most MASLD patients have steatosis (MASL) without necroinflammation or fibrosis requiring medical therapy [26].

In general, being overweight is a major risk factor for MASLD, which was estimated to account for 39% of adults aged 18 years and over in 2016 worldwide (who.int accessed on 9 June 2021). According to the Asia-Pacific criteria of the WHO guidelines for obesity (body mass index, BMI ≥ 25 kg/m^2^), Korean individuals with obesity and abdominal obesity reached 35.7% and 23.8%, respectively, in 2018 [27]. Ethnicity, sex and age are also important factors affecting the prevalence of MASLD [28]. The populations in Asian countries, including Korea, are known to be susceptible to nonobese MASLD [29]. A meta-analysis reported a MASH prevalence of 6.7% (in Asia) and 29.9% (in North America) among MASLD patients [30]. Both the numbers of newly diagnosed and treated patients with MASL and MASH peaked in the 60s in males and females in our results (Figure 3A–D). Regarding the sex-dimorphic feature of MASLD, our results showed male a predominance for those < 50 years old but a female predominance for those ≥ 50 years old for the incidence and prevalence of MASL and the incidence of MASH (Figure 3A–D). The menopausal age-specific feature in females is a known epidemiologic characteristic [31]. The reduced estrogen level causes a decline in choline synthesis and the hepatic export of very low-density lipoprotein (VLDL), leading to ectopic fat accumulation in hepatic tissue [32]. Globally, MASLD/MASH incidence increased 1.5-fold between 1990 and 2017 among children and young adults [33], while approximately 20% to 50% of young patients with MASLD had MASH at the time of diagnosis [34]. 

The burden of MASLD has become a public health issue worldwide, as it is not only a new causative disorder leading to liver-related death but also an independent risk factor for various extrahepatic disorders, such as obesity, type 2 diabetes, and cardiovascular diseases [4]. To date, many studies on the incidence or prevalence of MASLD have been performed, and those data came from relatively small populations with restricted ages or from subjects with certain disorders. We initially conducted an analysis of the national dataset encompassing patients with MASL and MASH across the entire Korean population. This study provides a genuine portrayal of the treatment landscape for patients with MASL and MASH within clinical settings, reflecting an increased awareness among physicians regarding the heightened risk associated with MASLD, particularly MASH.

## 5. Limitations

This study, however, has some limitations, which means we need a careful interpretation of its findings. Although approximately 97% of Koreans had health insurance coverage [35], there could be an exclusion from the MASLD data analyzed in this study. It is important to note that the present study was based on the available data within the Korean health insurance system, which would be different to those based on individual chart reviews for each patient. The lack of information for MASLD-related disorders, such as obesity, hyperlipidemia, and diabetes, is another limitation of the present study.

## 6. Conclusions

The present study first provides nationwide medically recorded data for MASLD in Korea and will serve as crucial reference data for the prevention and management of MASLD, including health-related policies. Further long-term studies investigating environmental factors and other possible contributing factors are needed to elucidate the increasing trend in the occurrence of MASLD.

## Figures and Tables

**Table 1:** Annual incidence (per 1000 persons) of MASL in Korea.

Year	Total			Male			Female		
	No. of Cases	Population	Incidence (95% CI)	No. of Cases	Population	Incidence (95% CI)	No. of Cases	Population	Incidence (95% CI)
2010	484,176	49,879,812	9.71 (9.68–9.73)	244,837	24,977,164	9.80 (9.76–9.84)	239,339	24,902,648	9.61 (9.57–9.65)
2011	490,054	50,111,476	9.78 (9.75–9.81)	253,077	25,081,788	10.09 (10.05–10.13)	236,977	25,029,688	9.47 (9.43–9.51)
2012	472,877	50,345,325	9.39 (9.37–9.42)	241,778	25,187,494	9.60 (9.56–9.64)	231,099	25,157,831	9.19 (9.15–9.22)
2013	471,125	50,558,952	9.32 (9.29–9.34)	238,193	25,282,928	9.42 (9.38–9.46)	232,932	25,276,024	9.22 (9.18–9.25)
2014	462,805	50,763,158	9.12 (9.09–9.14)	234,270	25,374,486	9.23 (9.20–9.27)	228,535	25,388,672	9.00 (8.96–9.04)
2015	484,644	50,951,719	9.51 (9.49–9.54)	245,839	25,458,058	9.66 (9.62–9.69)	238,805	25,493,662	9.37 (9.33–9.40)
2016	537,241	51,112,972	10.51 (10.48–10.54)	273,451	25,527,815	10.71 (10.67–10.75)	263,790	25,585,157	10.31 (10.27–10.35)
2017	542,305	51,230,704	10.59 (10.56–10.61)	274,920	25,576,752	10.75 (10.71–10.79)	267,385	25,653,952	10.42 (10.38–10.46)
2018	659,533	51,300,880	12.86 (12.83–12.89)	334,176	25,601,961	13.05 (13.01–13.10)	325,357	25,698,919	12.66 (12.62–12.70)
2019	750,324	51,337,424	14.62 (14.58–14.65)	379,509	25,609,342	14.82 (14.77–14.87)	370,815	25,728,082	14.41 (14.37–14.46)
2020	663,742	51,349,259	12.93 (12.90–12.96)	334,214	25,606,081	13.05 (13.01–13.10)	329,528	25,743,179	12.80 (12.76–12.84)
2021	716,188	51,333,253	13.95 (13.92–13.98)	362,871	25,589,102	14.18 (14.13–14.23)	353,317	25,744,151	13.72 (13.68–13.77)
Mean	561,251	50,856,244	11.02 (10.99–11.05)	284,761	25,406,081	11.20 (11.16–11.24)	276,490	25,450,163	10.85 (10.81–10.89)

CI, confidence interval.

**Table 2:** Annual incidence (per 1000 persons) of MASH in Korea.

Year	Total			Male			Female		
	No. of Cases	Population	Incidence (95%CI)	No. of Cases	Population	Incidence (95%CI)	No. of Cases	Population	Incidence (95%CI)
2010	18,519	49,879,812	0.37 (0.37–0.38)	9623	24,977,164	0.39 (0.38–0.39)	8896	24,902,648	0.36 (0.35–0.36)
2011	35,930	50,111,476	0.72 (0.71–0.72)	19,484	25,081,788	0.78 (0.77–0.79)	16,446	25,029,688	0.66 (0.65–0.67)
2012	42,687	50,345,325	0.85 (0.84–0.86)	23,139	25,187,494	0.92 (0.91–0.93)	19,548	25,157,831	0.78 (0.77–0.79)
2013	48,181	50,558,952	0.95 (0.94–0.96)	25,892	25,282,928	1.02 (1.01–1.04)	22,289	25,276,024	0.88 (0.87–0.89)
2014	49,428	50,763,158	0.97 (0.97–0.98)	26,775	25,374,486	1.06 (1.04–1.07)	22,653	25,388,672	0.89 (0.88–0.90)
2015	61,598	50,951,719	1.21 (1.20–1.22)	32,967	25,458,058	1.29 (1.28–1.31)	28,631	25,493,662	1.12 (1.11–1.14)
2016	77,686	51,112,972	1.52 (1.51–1.53)	41,285	25,527,815	1.62 (1.60–1.63)	36,401	25,585,157	1.42 (1.41–1.44)
2017	135,618	51,230,704	2.65 (2.63–2.66)	75,264	25,576,752	2.94 (2.92–2.96)	60,354	25,653,952	2.35 (2.33–2.37)
2018	227,007	51,300,880	4.43 (4.41–4.44)	123,114	25,601,961	4.81 (4.78–4.84)	103,893	25,698,919	4.04 (4.02–4.07)
2019	244,891	51,337,424	4.77 (4.75–4.79)	132,760	25,609,342	5.18 (5.16–5.21)	112,131	25,728,082	4.36 (4.33–4.38)
2020	256,764	51,349,259	5.00 (4.98–5.02)	138,733	25,606,081	5.42 (5.39–5.45)	118,031	25,743,179	4.58 (4.56–4.61)
2021	283,253	51,333,253	5.52 (5.50–5.54)	151,901	25,589,102	5.94 (5.91–5.97)	131,352	25,744,151	5.10 (5.07–5.13)
Mean	123,464	50,856,244	2.41 (2.40–2.43)	66,745	25,406,081	2.61 (2.60–2.63)	56,719	25,450,163	2.21 (2.20–2.23)

CI, confidence interval.

**Table 3:** Annual prevalence (per 1000 persons) of MASL in Korea.

Year	Total			Male			Female		
	No. of Cases	Population	Incidence (95% CI)	No. of Cases	Population	Incidence (95% CI)	No. of Cases	Population	Incidence (95% CI)
2010	782,815	49,879,812	15.69 (15.66–15.73)	403,786	24,977,164	16.17 (16.12–16.22)	379,029	24,902,648	15.22 (15.17–15.27)
2011	834,240	50,111,476	16.65 (16.61–16.68)	436,512	25,081,788	17.40 (17.35–17.45)	397,728	25,029,688	15.89 (15.84–15.94)
2012	859,439	50,345,325	17.07 (17.04–17.11)	446,978	25,187,494	17.75 (17.69–17.80)	412,461	25,157,831	16.39 (16.35–16.44)
2013	907,013	50,558,952	17.94 (17.90–17.98)	467,478	25,282,928	18.49 (18.44–18.54)	439,535	25,276,024	17.39 (17.34–17.44)
2014	948,609	50,763,158	18.69 (18.65–18.72)	486,199	25,374,486	19.16 (19.11–19.21)	462,410	25,388,672	18.21 (18.16–18.27)
2015	1,019,960	50,951,719	20.02 (19.98–20.06)	524,331	25,458,058	20.60 (20.54–20.65)	495,629	25,493,662	19.44 (19.39–19.49)
2016	1,142,133	51,112,972	22.35 (22.30–22.39)	588,331	25,527,815	23.05 (22.99–23.10)	553,802	25,585,157	21.65 (21.59–21.70)
2017	1,211,432	51,230,704	23.65 (23.60–23.69)	624,350	25,576,752	24.41 (24.35–24.47)	587,082	25,653,952	22.88 (22.83–22.94)
2018	1,396,198	51,300,880	27.22 (27.17–27.26)	712,367	25,601,961	27.82 (27.76–27.89)	683,831	25,698,919	26.61 (26.55–26.67)
2019	1,615,811	51,337,424	31.47 (31.43–31.52)	821,057	25,609,342	32.06 (31.99–32.13)	794,754	25,728,082	30.89 (30.82–30.96)
2020	1,603,009	51,349,259	31.22 (31.17–31.27)	811,650	25,606,081	31.70 (31.63–31.77)	791,359	25,743,179	30.74 (30.67–30.81)
2021	1,757,048	51,333,253	34.23 (34.18–34.28)	891,024	25,589,102	34.82 (34.75–34.89)	866,024	25,744,151	33.64 (33.57–33.71)
Mean	1,173,142	50,856,245	23.02 (22.97–23.06)	601,172	24,406,081	23.62 (23.56–23.68)	571,970	25,450,164	22.41 (22.36–22.47)

CI, confidence interval.

**Table 4:** Annual prevalence (per 1000 persons) of MASH in Korea.

Year	Total			Male			Female		
	No. of Cases	Population	Incidence (95% CI)	No. of Cases	Population	Incidence (95% CI)	No. of Cases	Population	Incidence (95% CI)
2010	24,496	49,879,812	0.49 (0.48–0.50)	12,863	24,977,164	0.51 (0.51–0.52)	11,633	24,902,648	0.47 (0.46–0.48)
2011	43,005	50,111,476	0.86 (0.85–0.87)	23,311	25,081,788	0.93 (0.92–0.94)	19,694	25,029,688	0.79 (0.78–0.80)
2012	55,921	50,345,325	1.11 (1.10–1.12)	30,541	25,187,494	1.21 (1.20–1.23)	25,380	25,157,831	1.01 (1.00–1.02)
2013	66,771	50,558,952	1.32 (1.31–1.33)	36,142	25,282,928	1.43 (1.41–1.44)	30,629	25,276,024	1.21 (1.20–1.23)
2014	73,447	50,763,158	1.45 (1.44–1.46)	39,679	25,374,486	1.56 (1.55–1.58)	33,768	25,388,672	1.33 (1.32–1.34)
2015	91,245	50,951,719	1.79 (1.78–1.80)	49,069	25,458,058	1.93 (1.91–1.94)	42,176	25,493,662	1.65 (1.64–1.67)
2016	115,232	51,112,972	2.25 (2.24–2.27)	61,654	25,527,815	2.42 (2.40–2.43)	53,578	25,585,157	2.09 (2.08–2.11)
2017	185,656	51,230,704	3.62 (3.61–3.64)	102,666	25,576,752	4.01 (3.99–4.04)	82,990	25,653,952	3.23 (3.21–3.26)
2018	316,035	51,300,880	6.16 (6.14–6.18)	173,851	25,601,961	6.79 (6.76–6.82)	142,184	25,698,919	5.53 (5.50–5.56)
2019	384,098	51,337,424	7.48 (7.46–7.51)	211,430	25,609,342	8.26 (8.22–8.29)	172,668	25,728,082	6.71 (6.68–6.74)
2020	432,276	51,349,259	8.42 (8.39–8.44)	237,555	25,606,081	9.28 (9.24–9.31)	194,721	25,743,179	7.56 (7.53–7.60)
2021	502,530	51,333,253	9.79 (9.76–9.82)	274,949	25,589,102	10.74 (10.70–10.78)	227,581	25,744,151	8.84 (8.80–8.88)
Mean	109,893	50,856,245	3.73 (3.71–3.74)	104,476	25,406,081	4.09 (4.07–4.11)	86,417	25,450,164	3.37 (3.35–3.39)

CI, confidence interval.

## Data Availability

The data used to support the findings of this study are included within the article including Supplementary Tables S1 and S2.

## Supplementary Materials

The following supporting information can be downloaded at: https://www.mdpi.com/article/10.3390/jcm12247634/s1, Table S1: Annual incidence (per 1000 persons) of MASL according to age group in Korea; Table S2: Annual incidence (per 1000 persons) of MASH according to age group in Korea.
