# Supplementary material for: The Clinical Diagnosis-Based Nationwide Epidemiology of Metabolic Dysfunction-Associated Liver Disease in Korea
Source: J Clin Med. 2023 Dec 12;12(24):7634. doi: 10.3390/jcm12247634 (PMC10744038; doi:10.3390/jcm12247634)
Supplement: Supplementary file 1 [file jcm-12-07634-s001.zip › jcm-2737171-supplementary.pdf]

Supplementary Tables

Table S1. Annual incidence (per 1000 persons) of MASL according to age group in Korea

Year	Incidence of Male/Female by age (year)							Total
	20-29	30-39	40-49	50-59	60-69	70-79	≥ 80	
2010	5.70	10.63	12.83	16.92	18.11	16.04	11.84	9.80
	5.78	8.10	10.53	18.39	20.01	16.52	9.13	9.61
2011	6.06	11.09	13.16	16.84	17.57	15.68	14.11	10.09
	5.64	7.90	10.27	17.75	18.76	16.04	10.01	9.47
2012	5.41	10.01	12.25	16.18	17.42	15.32	11.85	9.60
	5.19	7.31	9.76	17.10	18.39	15.70	9.54	9.19
2013	5.49	9.84	12.02	15.03	16.67	14.76	12.21	9.42
	5.29	7.31	9.75	16.81	18.12	15.01	9.43	9.22
2014	5.62	9.94	11.66	14.15	15.77	13.55	11.25	9.23
	5.33	7.39	9.28	15.99	17.17	14.15	9.29	9.00
2015	5.93	10.55	12.16	14.31	16.09	13.80	11.92	9.66
	5.39	7.49	9.52	16.43	17.83	14.52	9.59	9.37
2016	6.68	11.89	13.56	15.48	17.29	14.42	11.88	10.71
	5.92	8.42	10.36	18.04	19.31	15.22	9.76	10.31
2017	6.85	12.09	13.55	15.16	16.60	14.32	12.26	10.75
	6.26	8.61	10.50	17.69	18.77	15.01	10.25	10.42
2018	7.56	14.28	16.24	18.53	20.84	17.98	14.94	13.05
	6.76	9.94	12.28	21.43	23.80	19.02	12.13	12.66
2019	8.49	16.06	18.63	20.58	23.43	20.34	16.19	14.82
	7.54	11.20	14.11	23.55	26.90	21.85	13.49	14.41
2020	7.99	14.50	16.54	17.41	19.91	17.39	13.62	13.05
	7.23	10.49	12.62	19.95	23.21	18.72	11.56	12.80
2021	8.93	15.99	18.49	18.20	19.81	16.91	13.57	14.18
	8.30	12.09	14.12	20.72	23.24	18.35	11.21	13.72
Mean ± SD	6.73 ± 1.24*	12.24 ± 2.35*	14.26 ± 2.53*	16.57 ± 1.91*	18.29 ± 2.27*	15.88 ± 1.99*	12.97 ± 1.51*	11.20 ± 2.01*
	6.22 ± 1.02*	8.85 ± 1.66*	11.09 ± 1.74*	18.65 ± 2.29*	20.46 ± 3.06*	16.68 ± 2.32*	10.45 ± 1.36*	10.85 ± 1.98*

SD, standard deviation; * $p = 0.00$ according to Cochran-Armitage test for trend

Table S2. Annual incidence (per 1000 persons) of MASH according to age group in Korea

Year	Incidence of Male/Female by age (year)							Total
	20-29	30-39	40-49	50-59	60-69	70-79	≥ 80	
2010	0.23	0.37	0.43	0.63	0.77	0.80	1.02	0.39
	0.20	0.26	0.33	0.61	0.78	0.81	0.68	0.36
2011	0.49	0.86	0.95	1.20	1.32	1.31	1.49	0.78
	0.35	0.51	0.65	1.25	1.36	1.17	1.00	0.66
2012	0.59	1.07	1.14	1.33	1.45	1.45	1.44	0.92
	0.40	0.60	0.77	1.42	1.59	1.34	0.99	0.78
2013	0.65	1.20	1.24	1.39	1.62	1.47	1.30	1.02
	0.45	0.67	0.87	1.58	1.74	1.36	1.04	0.88
2014	0.67	1.25	1.31	1.45	1.60	1.45	1.30	1.06
	0.49	0.69	0.88	1.59	1.71	1.35	1.00	0.89
2015	0.82	1.53	1.59	1.76	2.02	1.90	1.94	1.29
	0.57	0.86	1.08	1.92	2.18	1.86	1.58	1.12
2016	1.06	1.98	2.09	2.13	2.34	2.03	2.04	1.62
	0.79	1.13	1.42	2.41	2.66	2.14	1.61	1.42
2017	1.64	3.30	3.98	4.22	4.50	3.74	3.40	2.94
	1.12	1.75	2.25	3.97	4.51	4.00	2.84	2.35
2018	2.71	5.52	6.48	6.65	7.32	6.24	5.58	4.81
	2.00	3.04	3.84	6.62	7.74	6.60	4.86	4.04
2019	3.24	6.35	7.05	6.63	7.32	6.35	5.77	5.18
	2.41	3.58	4.20	6.74	7.90	6.83	4.87	4.36
2020	3.52	6.64	7.44	6.83	7.48	6.40	5.74	5.42
	2.68	3.90	4.49	6.87	8.21	6.61	4.73	4.58
2021	4.03	7.30	8.14	7.12	7.58	6.45	5.70	5.94
	3.15	4.55	5.22	7.35	8.60	6.82	4.78	5.10
Mean ± SD	1.64 ± 1.36*	3.11 ± 2.59*	3.49 ± 2.95*	3.45 ± 2.63*	3.78 ± 2.84*	3.30 ± 2.37*	3.06 ± 2.04*	2.61 ± 2.12*
	1.22 ± 1.05*	1.80 ± 1.54*	2.17 ± 1.77*	3.53 ± 2.62*	4.08 ± 3.12*	3.41 ± 2.57*	2.50 ± 1.79*	2.21 ± 1.79*

SD, standard deviation; * $p = 0.00$ according to Cochran-Armitage test for trend
